# Pro- and Anti-Inflammatory Cytokines against Rv2031 Are Elevated during Latent Tuberculosis: A Study in Cohorts of Tuberculosis Patients, Household Contacts and Community Controls in an Endemic Setting

**DOI:** 10.1371/journal.pone.0124134

**Published:** 2015-04-21

**Authors:** Mulugeta Belay, Mengistu Legesse, Adane Mihret, Yonas Bekele, Tom H. M. Ottenhoff, Kees L. M. C. Franken, Gunnar Bjune, Fekadu Abebe

**Affiliations:** 1 Aklilu Lemma Institute of Pathobiology, Addis Ababa University, Addis Ababa, Ethiopia; 2 Department of Community Medicine, Institute of Health and Society, University of Oslo, Oslo, Norway; 3 Armauer Hansen Research Institute, Addis Ababa, Ethiopia; 4 Department of Infectious Diseases, Leiden University Medical Center, Leiden, The Netherlands; Fundació Institut d’Investigació en Ciències de la Salut Germans Trias i Pujol. Universitat Autònoma de Barcelona. CIBERES, SPAIN

## Abstract

Tuberculosis (TB) is among the leading causes of morbidity and mortality. The causative agent, *Mycobacterium tuberculosis* (*Mtb*), has evolved virulent factors for entry, survival, multiplication and immune evasion. Rv2031 (also called alpha crystallin, hspX, 16-kDa antigen), one of the most immunogenic latency antigens, is believed to play a key role in long-term viability of *Mtb*. Here, we report the dynamics of pro-inflammatory (IFN-γ, TNF-α) and anti-inflammatory (IL-10) cytokines against Rv2031 using whole blood assay in human cohorts in a TB endemic setting. Cytokine responses to ESAT-6-CFP-10 were also measured for comparison. Blood samples were collected from smear positive pulmonary TB patients and their contacts at baseline, 6 and 12 months, and from community controls at entry. At baseline, 54.4% of controls and 73.2% of contacts were QFT-GIT test positive. Baseline IFN-γ, TNF-α and IL-10 responses to Rv2031 were significantly higher in controls compared to contacts and untreated patients (p<0.001). Furthermore, untreated patients had significantly higher TNF-α and IL-10 responses to Rv2031 compared to contacts (p<0.001). In contacts and treated patients, IFN-γ, TNF-α and IL-10 responses to Rv2031 significantly increased over 12 months (p<0.0001) and became comparable with the corresponding levels in controls. There was a positive and significant correlation between Rv2031 and ESAT-6-CFP-10 specific cytokine responses in each study group. The fact that the levels of IFN-γ, TNF-α and IL-10 against Rv2031 were highest during latent TB infection may indicate their potential as markers of protection against TB. Taken together, the findings of this study suggest the potential of IFN-γ, TNF-α and IL-10 against Rv2031 as biomarkers of the host response to *Mtb* during convalescence from, and the absence of, active tuberculosis.

## Introduction

Tuberculosis (TB) remains a significant cause of morbidity and mortality worldwide with 9 million cases and 1.5 million deaths in 2013 alone [1]. Moreover, it is estimated that a third of the world population is infected with *Mycobacterium Tuberculosis (Mtb)* [2]. Such a high rate of latent TB infection implies the presence of a huge reservoir for active TB.

Bacille Calmette Guerin (BCG), the only available TB vaccine, has highly variable efficacy mainly limited to preventing severe forms of TB in children [3]. Therefore, there is an extensive research to develop an efficacious vaccine to replace or augment BCG [4]. Rv2031 (also known as alpha crystallin, hspX, 16-kDa) is among vaccine candidate proteins given increasing attention. Rv2031, an immunodominant protein, is one of the proteins expressed during latent TB infection. It was found to be the dominantly expressed protein in stationary phase and believed to play a key role in maintaining protein stability and long term viability of *Mtb* [5, 6]. The potential of this protein as a vaccine candidate has been investigated in several animal models [7–11] and encouraging results were reported. For example, adjunctive immunotherapy of mice with a DNA vaccine expressing Rv2031 was shown to significantly reduce bacillary load, shorten treatment duration, result in complete restoration of lung architecture and prevent reactivation [10]. Similarly, in guinea pigs, Rv2031-based vaccination was shown to impart protection against TB through enhanced production of IL-12, IL-10 and TGF-β [11].

A limited number of studies investigated IFN-γ response to RV2031 in humans and reported contradictory results. For example, a study from The Gambia [12] and Colombia [13] reported increased IFN-γ response to Rv2031 in household contacts compared to community controls whereas investigators of the VACSEL Study Group in Ethiopia and The Gambia [14] and another independent study from Ethiopia [15] reported significantly increased IFN-γ in community controls compared to both TB patients and contacts. Besides, previous reports were limited to IFN-γ response to Rv2031. However, in addition to IFN-γ, other cytokines, notably TNF-α, are considered to be essential for the control of TB infection [16, 17]. In addition, IL-10 is a regulatory cytokine believed to play a key role in suppressing inflammatory and immunopathological responses [16, 17] and hence contributing to the outcome of infection. We, therefore, investigated the dynamics of pro-inflammatory (IFN-γ, TNF-α) and anti-inflammatory (IL-10) cytokine responses to Rv2031 in TB patients (before and after treatment), their household contacts and community controls in a TB endemic setting. Results were compared with cytokine responses to ESAT-6-CFP-10 (E6C10) since these antigens are believed to be specific to *Mtb* complex. To the best of our knowledge, this is the first longitudinal study in human populations in an endemic setting, where pro-and anti-inflammatory cytokine responses to Rv2031 are compared in different cohorts.

## Materials and Methods

### Study area

This study was conducted in the capital of Ethiopia, Addis Ababa with estimated population of 2.7 million [18]. In Addis Ababa, the majority of TB patients are treated in health centers and therefore, 7 among 24 health centers (Akaki, Kotebe, Woreda 17, Kazanchis, Shiromeda, Teklehymanot and Woreda 9) with established Directly Observed Treatment, Short Course (DOTS) services for TB were included in this study.

### Study participants

From April to December 2012, smear positive pulmonary TB patients were recruited prospectively from selected health centers. According to the national guideline for TB, Leprosy and TB/HIV [19], patients with cough lasting more than 2 weeks are screened for TB. The diagnosis of smear positive pulmonary TB was made when at least two out of three consecutive sputum smear examinations were positive for acid fast bacilli. At the same time, we recruited household contacts living in the same house with pulmonary TB patients. Moreover, community controls were randomly selected and recruited from catchment areas of selected health centers and previous TB disease or contact with pulmonary TB patients was excluded by questionnaire.

Clinical assessment including weight, height and BCG scar examination was done for all participants. QuantiFERON-TB Gold In-Tube (QFT-GIT) test was used to screen contacts and controls for *Mtb* infection as previously described [20]. Besides, chest x-ray was used to screen for active TB in contacts and controls. Smear microscopy and culture were done for contacts with productive cough. All TB patients were treated with anti-TB drugs for 6 months. Based on the Ethiopian national guideline [19], contacts in this study were not offered prophylactic treatment for TB. Screening for HIV infection was done according to the national guideline [21] and only those without HIV infection were included. All participants were adults from 18–60 years; those with immunosuppressive conditions (pregnancy, diabetes and immunosuppressive therapy like steroids) were excluded. Patients and contacts were followed-up over 12 months with clinical examination and blood sample collection at entry, 6 and 12 months. In controls, blood samples were collected at entry. Baseline blood samples were collected from patients before they started treatment. Laboratory analysis was done at Armauer Hansen Research Institute (AHRI).

### Whole Blood Assay

Heparinized blood sample was diluted to a final concentration of 1:10 with RPMI 1640 medium containing L-glutamine (Sigma) supplemented with penicillin, 100 U/ml and streptomycin, 100 μg/ml (Sigma). E6C10 and Rv2031 were used to stimulate blood samples at a final concentration of 10 μg/ml. For positive and negative controls, instead of E6C10 and Rv2031, PHA at 10 μg/ml (Sigma) and RPMI 1640 media were used, respectively. After 48 hours of incubation at 37°C with 5% CO_2_, supernatants were harvested and stored at -80°C until ELISA was done.

### Cytokine ELISA

The levels of IFN-γ, TNF-α and IL-10 were measured using Ready-Set-Go! cytokine ELISA kits (eBioscience, USA). The detection limits of these kits were 4–500 pg/ml (IFN-γ and TNF-α) and 2–300 pg/ml (IL-10). ELISA was done according to the manufacturer’s instructions. Briefly, corning costar 96-well ELISA plates were coated overnight with anti-human IFN-γ, TNF-α, or IL-10 capture antibodies at 4°C. Plates were blocked with assay diluent for 1 hour at room temperature (RT). Standards and samples were added and incubated for 2 hours at RT. Assay diluent was added in two wells instead of samples to subtract for backgrounds. Detection antibodies were added and incubated for 1 hour at RT. This was followed by avidin-horseradish peroxidase incubation for 30 minutes at RT. Plates were washed 5 times with PBS containing 5% Tween 20 after each reaction; after enzyme incubation, plates were washed 7 times. Color was developed using 3, 3′, 5, 5′-tetramethylbenzidine substrate and after 15 minutes of incubation at RT, reaction was stopped using 2N sulfuric acid. Plates were read at 450 nm wave length and sample OD values were interpolated with a four-parameter curve-fit using the standards.

### Data analysis

Data points of negative control wells were subtracted from antigen stimulated data points to adjust for non-specific responses. Values below the detection limits of each cytokine were assumed to represent non-responders and set to ‘1’. Non-parametric tests were used to compare groups. Spearman’s correlation was used to investigate the correlation between E6C10 and Rv2031 specific cytokine responses. Kruskal-Wallis test with Dunn’s multiple comparisons was used to compare cytokine responses among TB patients, contacts and controls at baseline. Mann-Whitney U-test was used to compare cytokine responses between patients and contacts at 6 and 12 months. Freidman test with Dunn’s multiple comparisons was used to compare cytokine levels over 12 months in patients and contacts. P-values less than 0.05 were considered statistically significant. GraphPad Prism version 6.00 for Windows (GraphPad Software, La Jolla California USA, http://www.graphpad.com) was used for data analysis.

### Ethics statement

The study protocol was approved by the Institutional Review Board of Aklilu Lemma Institute of Pathobiology, the AHRI/ALERT Ethics Review Committee, the National Research Ethics Review Committee from Ethiopia and the Regional Committees for Medical and Health Research Ethics, South-East Norway (Regionale Komiteer for Medisinsk og Helsefaglig Forskningsetikk, Sør-Øst) from Norway. Written informed consent was obtained from each participant before inclusion into the study.

## Results

### Socio-demographic characteristics of study participants

A total of 147 smear positive pulmonary TB patients, 148 household contacts and 68 community controls were included in this study. There was no significant difference in the mean age and the proportion of those with BCG scar among the three groups. Besides, no significant associations between levels of cytokines and BCG scar were observed in all the groups. Patients, however, had significantly lower body mass index compared to both contacts and controls (P<0.001) (Table 1). In contacts, the median duration of exposure to TB patients was 12 (IQR: 4–24) weeks. Furthermore, the median daily exposure was 12 (IQR: 7.25–13) hours. In contacts, no significant association was observed between total or daily average duration of exposure to TB patients and QFT-GIT positivity. Twenty-one contacts reported previous exposure to a TB patient other than the patient included in the study. The proportion of QFT-GIT positivity was higher in those who reported previous exposure (86%) compared to those who had no known exposure to TB patients other than the index patient included in this study (70%) but this difference was not statistically significant (p = 0.26).

**Table 1:** Socio-demographic characteristics of study participants.

Characteristics	Patients	Contacts	Controls	p-value
Mean age in years	29.4	32.0	32.4	0.13
Proportion of males (%)	58.5	44.5	47.1	0.05
Mean BMI*	17.9	21.1	22.4	<0.001
Proportion with BCG scar (%)	28.1	37.0	35.3	0.36
QFT-GIT positive (%)	NA	73.2	54.4	0.005

* Mean body mass index (weight in kilograms divided by height in meters squared)

NA = data not available for all patients

### Cytokine responses to Rv2031 in contacts and controls stratified by QFT-GIT test result

At baseline, 54.4% and 73.2% of controls and contacts were QFT-GIT positive, respectively (Table 1). Rv2031-specific IFN-γ, TNF-α and IL-10 responses were comparable in QFT-GIT positive and negative contacts and controls. We, therefore, included all contacts and controls for further analysis.

### IFN-γ response to Rv2031 at baseline, 6 and 12 months

**Fig 1:**
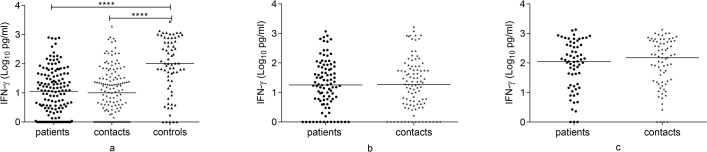
Comparison of IFN-γ response to Rv2031 among groups at baseline, 6 and 12 months. IFN-γ response at baseline (a), 6 months (b) and 12 months (c) following whole blood stimulation with Rv2031 for 48 hours. Filled circles represent patients, filled diamonds represent contacts and filled triangles represent controls. Results are individual responses and cytokine levels are expressed as log_10_ pg/ml. Horizontal bars are medians. Kruskal-Wallis test with Dunn’s multiple comparisons (a) and Mann-Whitney test (b and c) were used to compare groups. ****p<0.0001.

**Fig 2:**
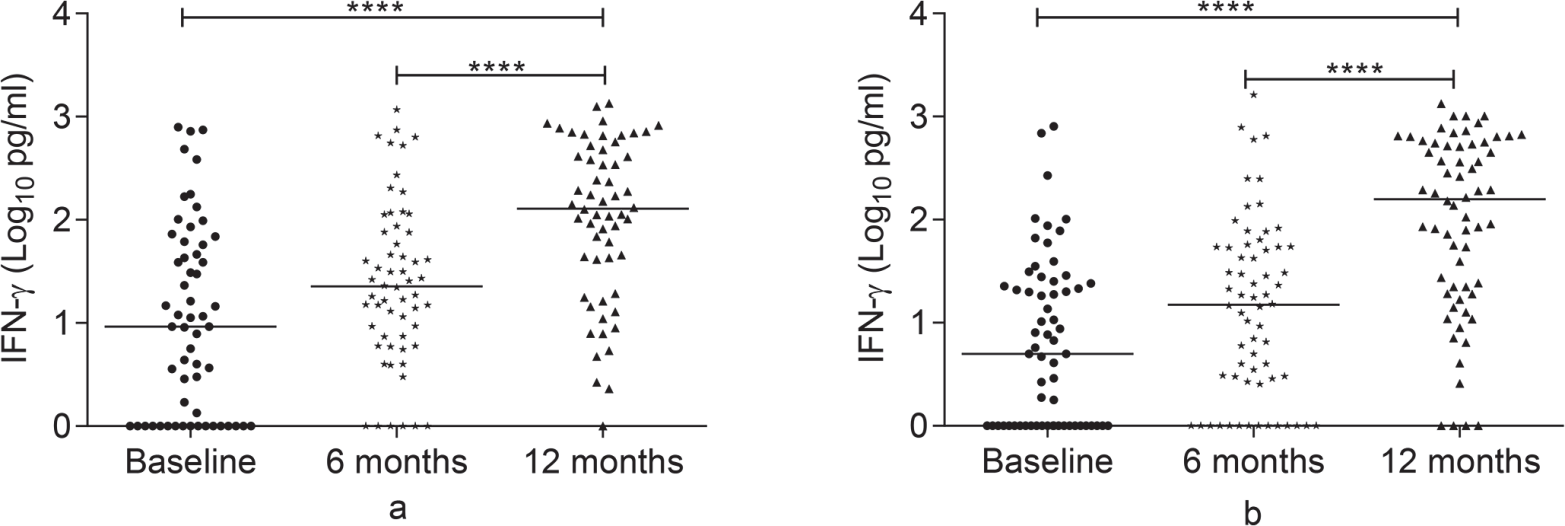
Changes in IFN-γ response to Rv2031 over 12 months in patients and contacts. IFN-γ response over 12 months in patients (a) and contacts (b) following whole blood stimulation with Rv2031 for 48 hours. Filled circles represent baseline levels, filled diamonds represent 6 month levels and filled triangles represent 12 month levels. Results are individual responses and cytokine levels are expressed as log_10_ pg/ml. Horizontal bars are medians. Freidman test with Dunn’s multiple comparisons was used to compare IFN-γ response over time. ****p<0.0001.

Figs 1 and 2 present IFN-γ response to Rv2031. At baseline, controls had a significantly higher IFN-γ response compared to contacts and untreated patients (P<0.0001). However, in contacts and treated patients, IFN-γ response significantly increased at 12 months compared to its levels at baseline and 6 months (p<0.0001).

### TNF-α response to Rv2031 at baseline, 6 and 12 months

**Fig 3:**
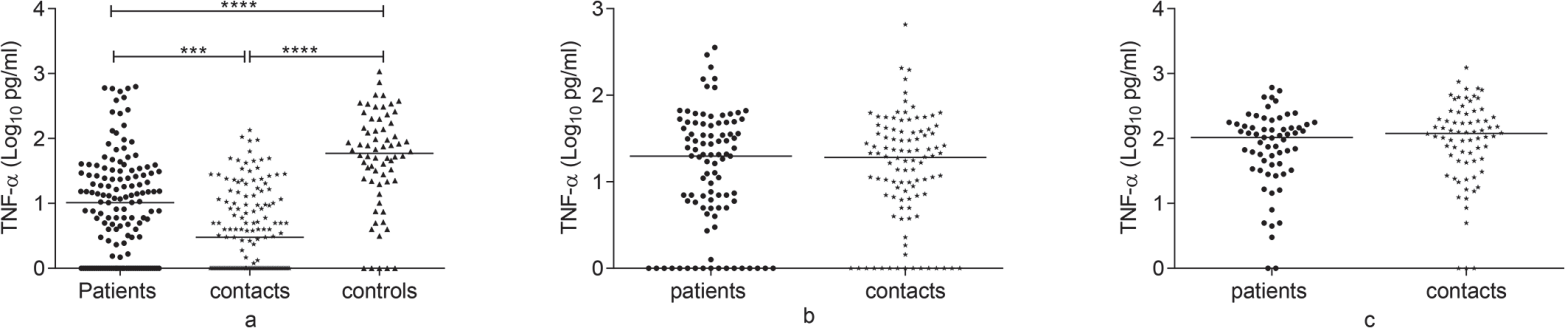
Comparison of TNF-α response to Rv2031 among groups at baseline, 6 and 12 months. TNF-α response at baseline (a), 6 months (b) and 12 months (c) following whole blood stimulation with Rv2031 for 48 hours. Filled circles represent patients, filled diamonds represent contacts and filled triangles represent controls. Results are individual responses and cytokine levels are expressed as log_10_ pg/ml. Horizontal bars are medians. Kruskal-Wallis test with Dunn’s multiple comparisons (a) and Mann-Whitney test (b and c) were used to compare groups. ***p<0.001, ****p<0.0001.

**Fig 4:**
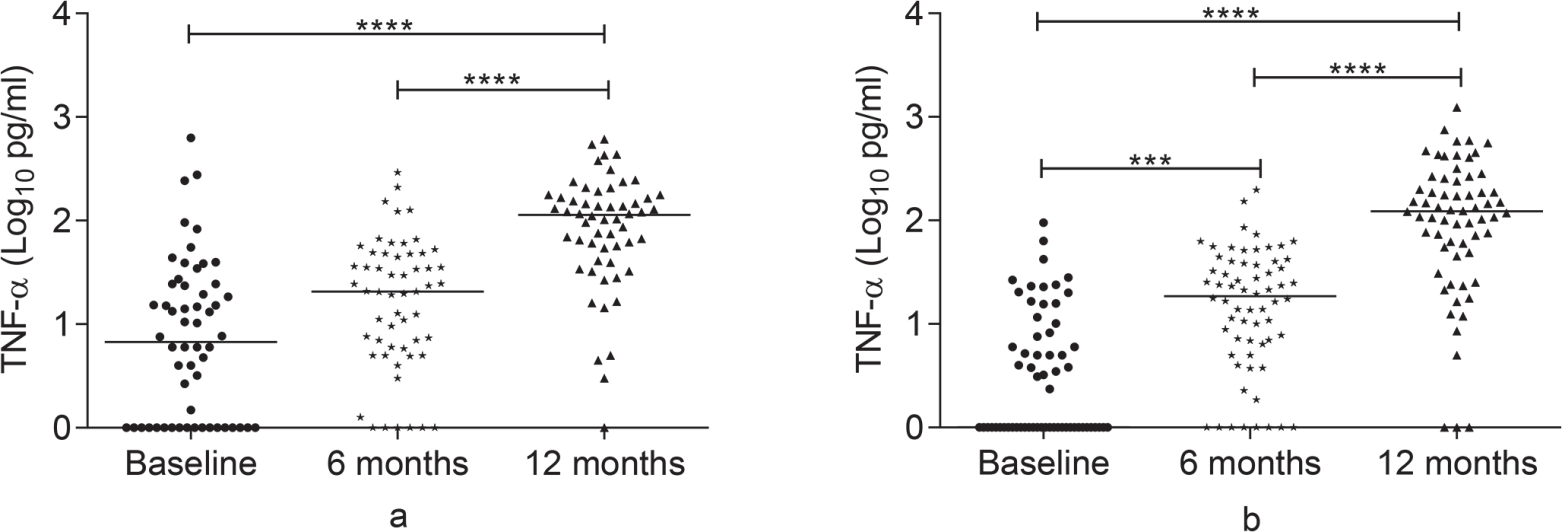
Changes in TNF-α response to Rv2031 over 12 months in patients and contacts. TNF-α response over 12 months in patients (a) and contacts (b) following whole blood stimulation with Rv2031 for 48 hours. Filled circles represent baseline levels, filled diamonds represent 6 month levels and filled triangles represent 12 month levels. Results are individual responses and cytokine levels are expressed as log_10_ pg/ml. Horizontal bars are medians. Freidman test with Dunn’s multiple comparisons was used to compare TNF-α response over time. ***p<0.001, ****p<0.0001.

TNF-α response to Rv2031 is summarized in Figs 3 and 4. At baseline, controls had a significantly higher TNF-α response to Rv2031 compared to contacts and untreated patients (p<0.0001). Besides, untreated patients had significantly higher TNF-α response compared to contacts (p<0.001). In treated patients, TNF-α response was significantly higher at 12 months compared to both baseline and 6 month levels (p<0.0001). In contacts, TNF-α response was significantly higher at 6 months compared to its baseline response (p<0.001) and at 12 months compared to both baseline and 6 month levels (p<0.0001).

### IL-10 response to Rv2031 at baseline, 6 and 12 months

**Fig 5:**
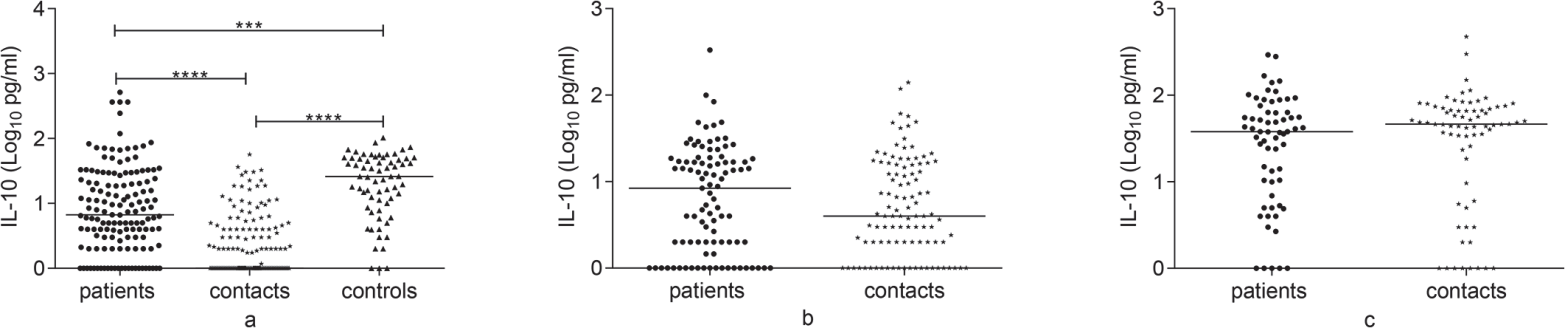
Comparison of IL-10 response to Rv2031 among groups at baseline, 6 and 12 months. IL-10 response at baseline (a), 6 months (b) and 12 months (c) following whole blood stimulation with Rv2031 for 48 hours. Filled circles represent patients, filled diamonds represent contacts and filled triangles represent controls. Results are individual responses and cytokine levels are expressed as log_10_ pg/ml. Horizontal bars are medians. Kruskal-Wallis test with Dunn’s multiple comparisons (a) and Mann-Whitney test (b and c) were used to compare groups. ***p<0.001, ****p<0.0001.

**Fig 6:**
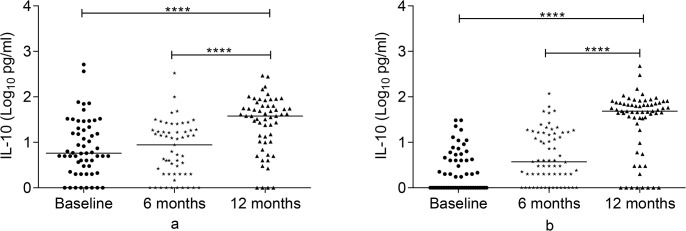
Changes in IL-10 responses to Rv2301 over 12 months in patients and contacts. IL-10 response over 12 months in patients (a) and contacts (b) following whole blood stimulation with Rv2031 for 48 hours. Filled circles represent baseline levels, filled diamonds represent 6 month levels and filled triangles represent 12 month levels. Results are individual responses and cytokine levels are expressed as log_10_ pg/ml. Horizontal bars are medians. Freidman test with Dunn’s multiple comparisons was used to compare IL-10 response over time. ****p<0.0001.

Figs 5 and 6 present IL-10 response to Rv2031. At baseline, controls had a significantly higher IL-10 response compared to untreated patients (p<0.001) and contacts (P<0.0001). Besides, untreated patients had significantly higher IL-10 response compared to contacts (p<0.0001). In contacts and treated patients, IL-10 significantly increased at 12 months compared to its level at baseline and 6 months (p<0.0001).

### Comparison of baseline IFN-γ/IL-10 and TNF-α/IL-10 ratios among groups

**Fig 7:**
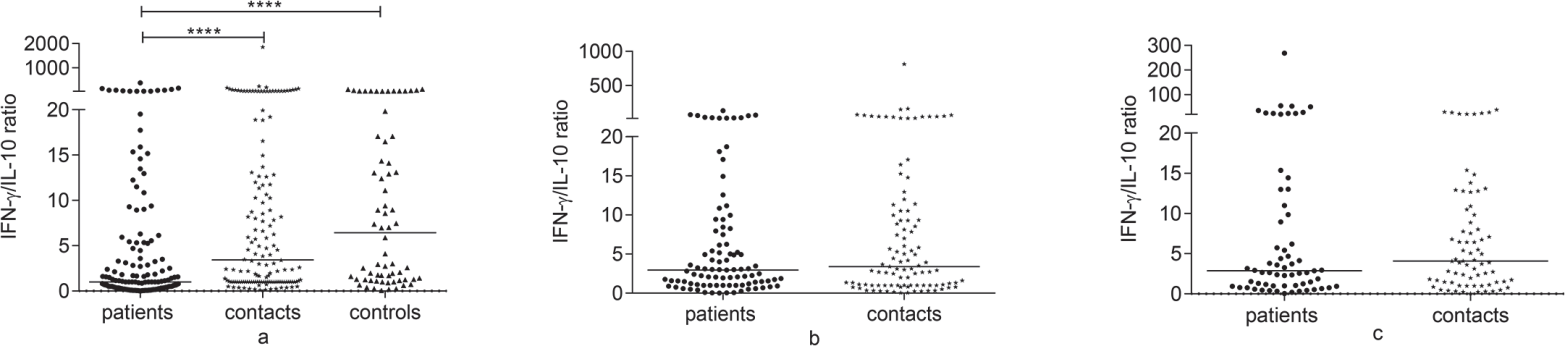
Comparison of IFN-γ/IL-10 ratio among groups at baseline, 6 and 12 months. IFN-γ/IL-10 ratio at baseline (a), 6 months (b) and 12 months (c) following whole blood stimulation with Rv2031 for 48 hours. Filled circles represent patients, filled diamonds represent contacts and filled triangles represent controls. Results are individual responses. Horizontal bars are medians. Kruskal-Wallis test with Dunn’s multiple comparisons (a) and Mann-Whitney test (b and C) were used to compare groups. ****p<0.0001.

**Fig 8:**
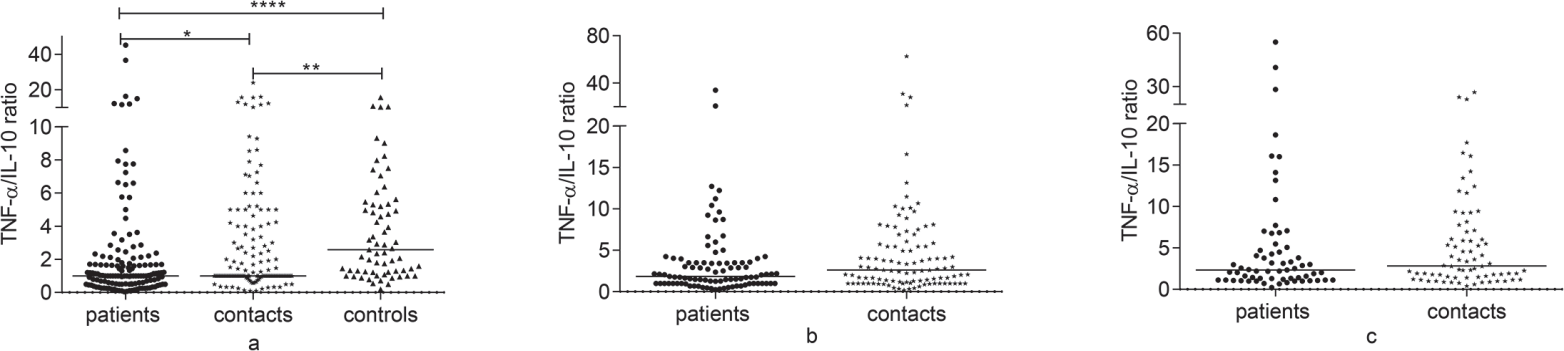
Comparison of TNF-α/IL-10 ratio among groups at baseline, 6 and 12 months. TNF-α/IL-10 ratio at baseline (a), 6 months (b) and 12 months (c) following whole blood stimulation with Rv2031 for 48 hours. Filled circles represent patients, filled diamonds represent contacts and filled triangles represent controls. Results are individual responses. Horizontal bars are medians. Kruskal-Wallis test with Dunn’s multiple comparisons (a) and Mann-Whitney test (b and C) were used to compare groups. *p = 0.03, **p<0.01, ****p<0.0001.

Baseline IFN-γ/IL-10 ratio was significantly lower in untreated patients compared to both contacts and controls (p<0.0001) (Fig 7). Similarly, baseline TNF-α/IL-10 ratio was significantly lower in untreated patients compared to contacts (p = 0.03) and controls (p<0.0001). Besides, controls had higher TNF-α/IL-10 ratio compared to contacts (p<0.01) (Fig 8). In contacts and treated patients, IFN-γ/IL-10 and TNF-α/IL-10 ratios increased over 12 months and the increase in TNF-α/IL-10 ratio was significant in both groups (p<0.01) (data not shown).

### IFN-γ, TNF-α and IL-10 responses to E6C10 at baseline, 6 and 12 months

**Fig 9:**
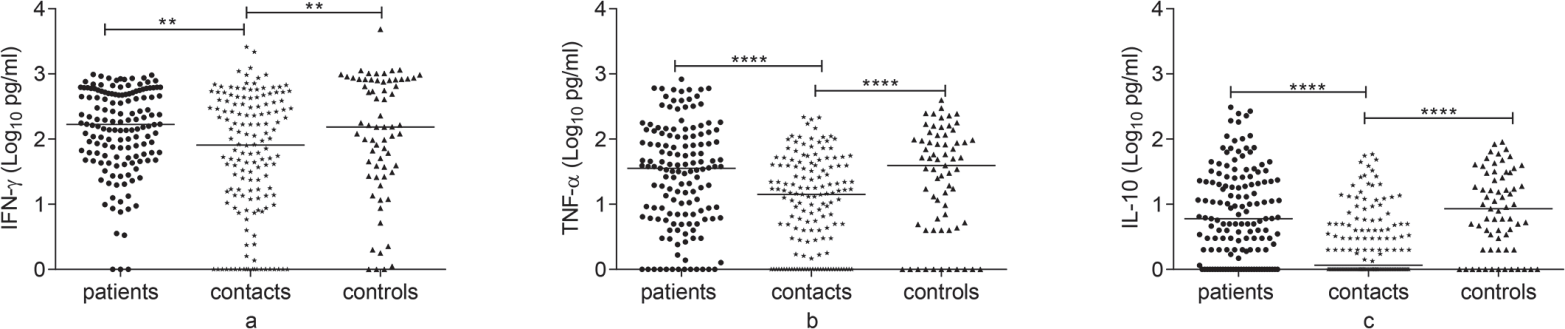
Comparison of baseline cytokine responses to E6C10 among groups. Baseline IFN-γ (a), TNF-**α** (b) and IL-10 (c) responses to E6C10 in patients, contacts and controls following whole blood stimulation for 48 hours. Filled circles represent patients, filled diamonds represent contacts and filled triangles represent controls. Results are individual responses and cytokine levels are expressed as log_10_ pg/ml. Horizontal bars are medians. Kruskal-Wallis test with Dunn’s multiple comparisons was used to compare groups. **p<0.01, ****p<0.0001.

**Fig 10:**
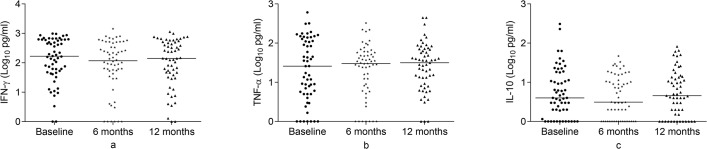
Changes in cytokine responses to E6C10 over 12 months in patients. IFN-γ (a), TNF-**α** (b) and IL-10 (c) responses to E6C10 in patients at baseline, 6 and 12 months following whole blood stimulation with E6C10 for 48 hours. Filled circles represent baseline levels, filled diamonds represent 6 month levels and filled triangles represent 12 month levels. Results are individual responses and cytokine levels are expressed as log_10_ pg/ml. Horizontal bars are medians. Freidman test with Dunn’s multiple comparisons was used to compare cytokine levels overtime.

**Fig 11:**
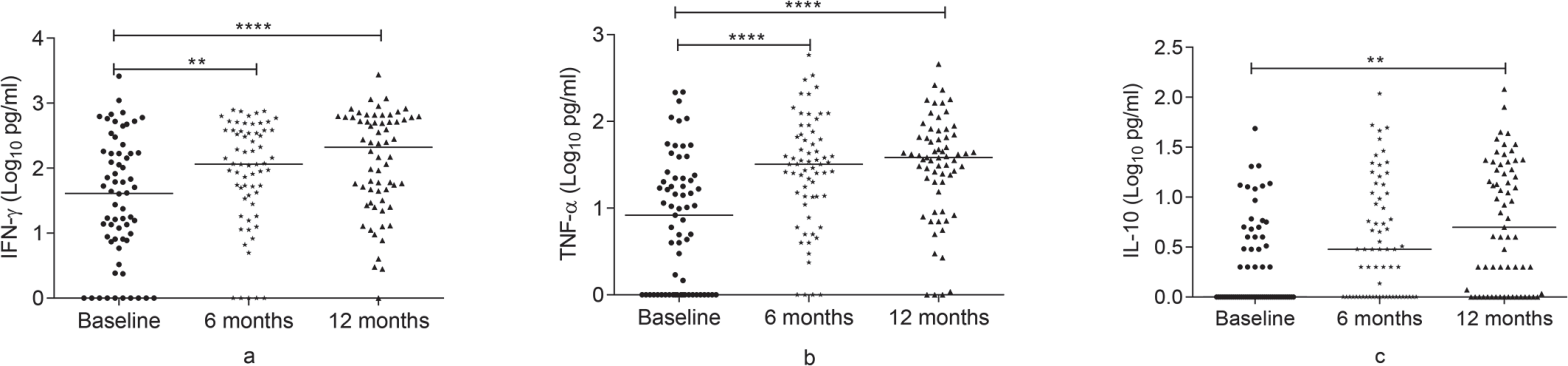
Changes in cytokine responses to E6C10 over 12 months in contacts. IFN-γ (a), TNF-**α** (b) and IL-10 (c) responses to E6C10 in contacts at baseline, 6 and 12 months following whole blood stimulation with E6C10 for 48 hours. Filled circles represent baseline levels, filled diamonds represent 6 month levels and filled triangles represent 12 month levels. Results are individual responses and cytokine levels are expressed as log_10_ pg/ml. Horizontal bars are medians. Freidman test with Dunn’s multiple comparisons was used to compare cytokine levels overtime. **p<0.01, ****p<0.0001.

At baseline, contacts had significantly lower IFN-γ, TNF-**α** and IL-10 compared to both untreated patients and controls (p<0.01) whereas untreated patients and controls had comparable cytokine levels (Fig 9). No significant change in the cytokine response to E6C10 was observed in patients after treatment (Fig 10); in contrast, contacts had significantly increased IFN-γ, TNF-**α** and IL-10 responses at 6 and 12 months compared to the corresponding baseline responses (p<0.01) (Fig 11).

### Correlation between E6C10 and Rv2031 induced cytokines

Correlations between E6C10 and Rv2031 specific cytokine responses were analyzed at baseline and there was a significant and positive correlation for each cytokine in the three groups (Table 2). The correlation for each cytokine in controls was relatively strong. Besides, the correlation for IFN-γ as well as IL-10 remained significant at 6 and 12 months but for TNF-**α**, there was no significant correlation at 12 months both in patients and contacts.

**Table 2:** Correlation between E6C10 and Rv2031 specific IFN-γ, TNF-α and IL-10.

	Patients	Contacts	Controls
**IFN-γ** (Rv2031 vs. E6C10)			
Baseline	0.36**	0.41**	0.65**
6 months	0.35**	0.36**	
12 months	0.43**	0.37**	
**TNF-α** (Rv2031 vs. E6C10)			
Baseline	0.44**	0.34**	0.79**
6 months	0.59**	0.41**	
12 months	0.19	0.18	
**Il-10** (Rv2031 vs. E6C10)			
Baseline	0.53**	0.62**	0.70**
6 months	0.69**	0.63**	
12 months	0.36**	0.54**	

^Vs.^ versus

**p<0.01

For each group, the correlation between E6C10 and Rv2031 specific IFN-γ, TNF-α and IL-10 responses were investigated using Spearman’s correlation and at baseline, Rv2031-specific cytokine responses were positively and significantly correlated with the corresponding cytokine responses to E6C10.

## Discussion

In this study, we showed that the levels of both pro-(IFN-γ, TNF-α) and anti-inflammatory (IL-10) cytokines against the latency antigen Rv2031 were significantly higher in community controls compared to untreated TB patients and their contacts. Furthermore, in patients and contacts, all cytokines significantly increased at 12 months and became comparable with the corresponding levels in controls.

In this study, controls had a strong Rv2031-specific IFN-γ response in agreement with a previous observation from Ethiopia and The Gambia [14]. Similarly, in a non-endemic setting, previously exposed contacts had strong IFN-γ response to latency antigens compared to untreated TB patients [22]. On the other hand, investigators from The Netherlands found comparable IFN-γ response to Rv2031 in untreated TB patients and individuals with latent TB infection [23]; however, it is not clear if the Dutch study included those with recent or past exposure. In our study, both recently exposed contacts and untreated TB patients had low IFN-γ response to Rv2031. In endemic settings, considering the high exposure and TB infection rates, community controls could safely be assumed to have a protective immunity as opposed to untreated TB patients who failed to control the infection [14] and therefore, a high IFN-γ response to Rv2031 in controls is suggestive of protective immunity against TB in this group. In addition to increased IFN-γ response as previously reported [14], we observed a significantly higher baseline TNF-α and IL-10 responses to Rv2031 in controls compared to untreated patients and contacts. These data further suggest that cytokines other than IFN-γ are induced by Rv2031 during latent TB infection and may contribute towards protection against progression of TB infection. Moreover, we have measured IFN-γ, TNF-α and Il-10 responses against E6C10, antigens believed to be specific to *Mtb* complex, for comparison and the results showed a significant and positive correlation between E6C10 and Rv2031 specific cytokine responses especially in controls suggesting that cytokine responses to Rv2031 are *Mtb* complex specific.

Put together, our data suggest that IFN-γ, TNF-α and IL-10 against Rv2031 could be potential biomarkers of protective immunity in those with latent TB infection. Furthermore, in a mouse model, it was observed that long term protection following vaccination with BCG overexpressing Rv2031 [9] and DNA containing Rv2031 [24] were associated with a significant increase in IFN-γ response to Rv2031. Hence, our data together with previous observations in humans and animal models suggest the potential of Rv2031 as a vaccine candidate.

Contacts and treated patients had a significant increase in the levels of Rv2031 specific IFN-γ, TNF-α and IL-10 over 12 months. In agreement with our observation for IFN-γ, it was previously reported that untreated TB patients had low Rv2031 specific IFN-γ response followed by a significant increase after chemotherapy [25]. Similarly, in a murine model, investigators observed increased IFN-γ response to Rv2031 in the later stages of infection [8]. The low cytokine response during clinical TB followed by a strong cytokine response after chemotherapy in patients suggests the recovery of the immune response accompanied by bacterial reduction and this could further augment infection control and probably prevent relapse. On the other hand, the low cytokine response in contacts during early infection followed by a strong cytokine response after 6 months without prophylactic treatment may suggest the transition to latent *Mtb* infection with reduction of *bacteria* and development of protective immunity. Besides, recent infection, at least in some of the contacts, might account for low cytokine response. Our data suggest that cytokine response to Rv2031 is dynamic with low response in recent exposure/infection or clinical TB followed by increased cytokine levels probably associated with increased expression of Rv2031 during latent TB infection [5]. Supporting this notion, a concurrent increase in the expression of Th1 immunity and Rv2031 together with other latency antigens was observed in a mouse model [26]. Rv2031 is considered to be an important antigen that maintains protein stability and hence long term viability of *Mtb* in macrophages [5, 27] and its increased expression during latency might be crucial for *Mtb* survival.

Further analysis of cytokine ratios revealed a stage-specific trend in which both IFN-γ/IL-10 and TNF-α/IL-10 ratios were lowest in untreated patients and highest in controls with a significant difference among the three groups. Although TNF-α and IL-10 were significantly higher in patients compared to contacts, TNF-α/IL-10 ratio was significantly lower in patients compared to contacts. Besides, IFN-γ/IL-10 and TNF-α/IL-10 ratios increased over time in treated patients and contacts. Taken together, these data suggest that apart from the absolute amount of each cytokine, the balance between pro-inflammatory (IFN-γ and TNF-α) and anti-inflammatory (IL-10) cytokine responses to Rv2031 could determine the outcome of infection [16].

We did not find any significant difference in the immune response to Rv2031 in QFT-GIT positive and negative contacts and controls. This might be because the infection is either too early to develop cytokine response to Rv2031 (contacts) or too remote to be detected by QFT-GIT (controls). Supporting the later assertion, reversions to QFT-GIT negative in QFT-GIT positive healthy individuals over time has been reported as a common event [28, 29]. In addition, the strong immune response in controls in this study seems unlikely to be related to previous BCG vaccination as our data suggests. Generally, immune response to BCG wanes after 15 years and all our study participants were adults. Moreover, whereas one study reported a strong IFN-γ response in BCG vaccinees [25], a minimal T-cell response against Rv2031 in BCG vaccinated population was observed in another study [23]. Similarly, a study from The Gambia reported lack of IFN-γ response in BCG vaccinated infants [12].

The present study showed that during natural *Mtb* infection in humans in endemic settings, contacts with recent exposure and /or infection and patients with clinical TB have low baseline cytokine response to Rv2031 followed by a significant and sustained increase of both pro- and anti-inflammatory cytokines after 6 months. A high IFN-γ, TNF-α and IL-10 against Rv2031 during latent TB infection suggests their role as protective markers of TB in humans. Taken together, the findings of this study suggest the potential of IFN-γ, TNF-α and IL-10 against Rv2031 as biomarkers of the host response to *Mtb* during convalescence from, and the absence of, active tuberculosis.

## References

[1] WHO. Global Tuberculosis Report 2014. WHO, Geneva; 2014 Available: http://www.who.int/tb/publications/global_report/en/.

[2] Manabe YC, Bishai WR. Latent *Mycobacterium tuberculosis*-persistence, patience, and winning by waiting. Nat Med. 2000; 6(12):1327–1329. 11100115 10.1038/82139

[3] Colditz GA, Brewer TF, Berkey CS, Wilson ME, Burdick E, Fineberg HV, et al Efficacy of BCG vaccine in the prevention of tuberculosis. Meta-analysis of the published literature. JAMA. 1994; 271(9):698–702. 8309034

[4] Abebe F. Is interferon-gamma the right marker for bacille Calmette-Guerin-induced immune protection? The missing link in our understanding of tuberculosis immunology. Clin Exp Immunol. 2012; 169(3):213–219. 10.1111/j.1365-2249.2012.04614.x 22861360 PMC3444997

[5] Yuan Y, Crane DD, Barry CE 3rd. Stationary phase-associated protein expression in *Mycobacterium tuberculosis*: function of the mycobacterial alpha-crystallin homolog. J Bacteriol. 1996; 178(15):4484–4492. 8755875 10.1128/jb.178.15.4484-4492.1996 PMC178214

[6] Yuan Y, Crane DD, Simpson RM, Zhu YQ, Hickey MJ, Sherman DR, et al The 16-kDa alpha-crystallin (Acr) protein of *Mycobacterium tuberculosis* is required for growth in macrophages. Proc Natl Acad Sci USA. 1998; 95(16):9578–9583. 9689123 10.1073/pnas.95.16.9578 PMC21381

[7] Dey B, Jain R, Gupta UD, Katoch VM, Ramanathan VD, Tyagi AK. A booster vaccine expressing a latency-associated antigen augments BCG induced immunity and confers enhanced protection against tuberculosis. PLoS One. 2011; 6(8):e23360 10.1371/journal.pone.0023360 21858087 PMC3157374

[8] Jeon BY, Kim SC, Eum SY, Cho SN. The immunity and protective effects of antigen 85A and heat-shock protein X against progressive tuberculosis. Microbes Infect.2011; 13(3):284–290. 10.1016/j.micinf.2010.11.002 21093603

[9] Shi C, Chen L, Chen Z, Zhang Y, Zhou Z, Lu J, et al Enhanced protection against tuberculosis by vaccination with recombinant BCG over-expressing HspX protein. Vaccine. 2010; 28(32):5237–5244. 10.1016/j.vaccine.2010.05.063 20538090

[10] Chauhan P, Jain R, Dey B, Tyagi AK. Adjunctive immunotherapy with alpha-crystallin based DNA vaccination reduces tuberculosis chemotherapy period in chronically infected mice. Sci Rep. 2013; 3:1821 10.1038/srep01821 23660989 PMC3650662

[11] Dey B, Jain R, Khera A, Gupta UD, Katoch VM, Ramanathan VD, et al Latency antigen alpha-crystallin based vaccination imparts a robust protection against TB by modulating the dynamics of pulmonary cytokines. PLoS One. 2011; 6(4):e18773 10.1371/journal.pone.0018773 21533158 PMC3078913

[12] Vekemans J, Ota MO, Sillah J, Fielding K, Alderson MR, Skeiky YA, et al Immune responses to mycobacterial antigens in the Gambian population: implications for vaccines and immunodiagnostic test design. Infect Immun. 2004; 72(1):381–388. 14688119 10.1128/IAI.72.1.381-388.2004 PMC343957

[13] del Corral H, Paris SC, Marin ND, Marin DM, Lopez L, Henao HM, et al IFN gamma response to *Mycobacterium tuberculosis*, risk of infection and disease in household contacts of tuberculosis patients in Colombia. PLoS One. 2009; 4(12):e8257 10.1371/journal.pone.0008257 20011589 PMC2788133

[14] Demissie A, Leyten EM, Abebe M, Wassie L, Aseffa A, Abate G, et al Recognition of stage-specific mycobacterial antigens differentiates between acute and latent infections with *Mycobacterium tuberculosis* . Clin Vaccine Immunol. 2006; 13(2):179–186. 16467323 10.1128/CVI.13.2.179-186.2006 PMC1391929

[15] Mamo G, Mihret A, Taffesse M, Gebru G, Afework M, Yamuah LK, et al T cell response to alpha crystallin and *Mycobacterium tuberculosis* specific antigens using ex-vivo elispot assay for detecting latent tuberculosis infection in Addis Ababa, Ethiopia. Ethiop Med J. 2014; Suppl 1:15–22. 24696984

[16] Cavalcanti YV, Brelaz MC, Neves JK, Ferraz JC, Pereira VR. Role of TNF-Alpha, IFN-Gamma, and IL-10 in the development of pulmonary tuberculosis. *Pulmon Med*. 2012:745483. 10.1155/2012/745483 PMC3515941 23251798

[17] Raja A. Immunology of tuberculosis. Indian J Med Res. 2004; 120(4):213–232. 15520479

[18] Population Census Commission. Summary and statistical report of the 2007 population and housing census: population size by age and sex CSA, Addis Ababa; 2007 Available: http://ecastats.uneca.org/aicmd/Portals/0/Cen2007_firstdraft.pdf.

[19] MOH. Guidelines for clinical and programmatic management of TB, Leprosy and TB/HIV in Ethiopia Ministry of Health, Addis Ababa; 2012 Available: http://www.medbox.org/ethiopia/guidelines-for-clinical-and-programmatic-management-of-tb-tbhiv-and-leprosy-in-ethiopia/preview.

[20] Belay M, Legesse M, Dagne D, Mihret A, Bekele Y, Medhin G, et al QuantiFERON-TB Gold In-Tube test conversions and reversions among tuberculosis patients and their household contacts in Addis Ababa: a one year follow-up study. BMC Infect Dis. 2014; 14(1):654 25466365 10.1186/s12879-014-0654-5 PMC4264256

[21] MOH. Guidelines for HIV counselling and testing in Ethiopia Ministry of Health, Addis Ababa; 2007 Available: http://www.who.int/hiv/topics/vct/ETH_HCT_guidelinesJune26_clean.pdf.

[22] Leyten EM, Lin MY, Franken KL, Friggen AH, Prins C, van Meijgaarden KE, et al Human T-cell responses to 25 novel antigens encoded by genes of the dormancy regulon of *Mycobacterium tuberculosis* . Microbes Infect. 2006; 8(8):2052–2060. 16931093 10.1016/j.micinf.2006.03.018

[23] Geluk A, Lin MY, van Meijgaarden KE, Leyten EM, Franken KL, Ottenhoff TH, et al T-cell recognition of the HspX protein of *Mycobacterium tuberculosis* correlates with latent M. tuberculosis infection but not with M. bovis BCG vaccination. Infect Immun.2007; 75(6):2914–2921. 17387166 10.1128/IAI.01990-06 PMC1932904

[24] Roupie V, Romano M, Zhang L, Korf H, Lin MY, Franken KL, et al Immunogenicity of eight dormancy regulon-encoded proteins of *Mycobacterium tuberculosis* in DNA-vaccinated and tuberculosis-infected mice. Infect Immun. 2007; 75(2):941–949. 17145953 10.1128/IAI.01137-06 PMC1828490

[25] Wilkinson RJ, Wilkinson KA, De Smet KA, Haslov K, Pasvol G, Singh M, et al Human T- and B-cell reactivity to the 16kDa alpha-crystallin protein of *Mycobacterium tuberculosis* . Scand J Immunol. 1998; 48(4):403–409. 9790311 10.1046/j.1365-3083.1998.00420.x

[26] Shi L, Jung YJ, Tyagi S, Gennaro ML, North RJ. Expression of Th1-mediated immunity in mouse lungs induces a *Mycobacterium tuberculosis* transcription pattern characteristic of nonreplicating persistence. Proc Natl Acad Sci USA. 2003; 100(1):241–246. 12506197 10.1073/pnas.0136863100 PMC140939

[27] Honer zu Bentrup K, Russell DG. Mycobacterial persistence: adaptation to a changing environment. Trends Microbiol. 2001; 9(12):597–605. 11728873 10.1016/s0966-842x(01)02238-7

[28] Pai M, Joshi R, Dogra S, Zwerling AA, Gajalakshmi D, Goswami K, et al T-cell assay conversions and reversions among household contacts of tuberculosis patients in rural India. Int J Tuberc Lung Dis. 2009; 13(1):84–92. 19105884 PMC2951989

[29] Ringshausen FC, Nienhaus A, Schablon A, Schlosser S, Schultze-Werninghaus G, Rohde G. Predictors of persistently positive *Mycobacterium-tuberculosis*-specific interferon-gamma responses in the serial testing of health care workers. BMC Infect Dis. 2010; 10:220 10.1186/1471-2334-10-220 20653946 PMC2916913

